# Hepatitis B virus genotype is an independent prognostic factor of telbivudine and tenofovir treatment in hepatitis B surface antigen‐positive pregnant women

**DOI:** 10.1002/fsn3.2619

**Published:** 2021-11-29

**Authors:** Baofang Zhang, Lei Yu, Mingliang Cheng, Quan Zhang, Jun Wu, Jing Yang, Qin Liu, Shuang Lu, Xueke Zhao, KaiSheng Deng, Yongmei Liu, Jun Wang, Peiling Zhao

**Affiliations:** ^1^ The Affliated Hospital Guizhou Medical University Guiyang, Guizhou China; ^2^ Prenatal Diagnosis Center Guizhou Medical University Guiyang, Guizhou China; ^3^ Laboratory Guizhou Medical University Guiyang, Guizhou China; ^4^ Clinical Research Center Guizhou Medical University Guiyang, Guizhou China

**Keywords:** genotype, hepatitis B virus, pregnancy, telbivudine, tenofovir

## Abstract

To investigate whether HBV genotype influences the effect of tenofovir and telbivudine on HBV DNA and RNA levels in HBsAg‐positive pregnant women. This was a retrospective study of 74 HBsAg‐positive pregnant women in Guizhou of China. All patients were treated with telbivudine or tenofovir from 12 weeks of pregnancy and HBV infection to the date of delivery. Blood samples were collected at 12–24, 28–32, and 36–40 weeks of pregnancy for the measurement of genotype, HBsAg, hepatitis B e antigen (HBeAg), HBV DNA, HBV RNA, and liver function, including alanine transaminase, aspartate transaminase, total bilirubin, total bile acids, cholinesterase, alkaline phosphatase (ALP), and gamma‐glutamyl transferase. All women with HBsAg were followed up. The HBV genotype was B in 64.9% and C in 35.1%. There were 37 patients of telbivudine and tenofovir group respectively. The telbivudine and tenofovir groups showed no differences in demographic and clinical characteristics, including liver function tests, HBsAg, HBeAg, log_10_(HBV DNA), and log_10_(HBV RNA). Compared with baseline (12–24 weeks), telbivudine group showed a significant increase in ALP and significant reductions in HBsAg, HBeAg, log_10_(HBV DNA), and log_10_(HBV RNA) at 36–40 weeks (*p* < .05). Tenofovir group exhibited a significant increase in ALP and significant reductions in HBeAg, log_10_(HBV DNA), and log_10_(HBV RNA) at 36–40 weeks, compared with baseline (*p* < .05). HBV genotype (B vs. C) was independently associated with HBV DNA change after therapy (*p* = .005). In telbivudine group, log_10_ (HBV DNA) increased from 3.38 (2.00–7.30) to 7.43 (4.68–8.70). In tenofovir group, log_10_ (HBV DNA) decreased from 7.52 (3.32–8.70) to 2.98 (2.00–5.01). HBV genotype was independently associated with HBV DNA change response to telbivudine or tenofovir in pregnant women with hepatitis B. These findings might be helpful for risk assessment regarding vertical transmission of HBV in HBeAg‐positive mothers treated with nucleos(t)ide analogues.

## 1 INTRODUCTION

Hepatitis B virus (HBV) infection is considered as one of the major public health problems. Serologic studies have indicated that around 2 billion people worldwide have been infected with HBV and more than 350 million people are chronic HBV carriers (Schmit et al., 2021; Trépo et al., 2014). Chronic hepatitis B (CHB) is an important risk factor for the development of liver cirrhosis and hepatocellular carcinoma, which has placed a heavy burden on patients, families, and society.

Indeed, it has been estimated that 40% men and 15% women with perinatally acquired HBV infection will die of liver cirrhosis or cancer. An epidemiologic survey conducted in China in 2014 revealed that the prevalence of hepatitis B surface antigen (HBsAg) positivity was 0.3% in children aged 1–4 years, 0.9% in children aged 5–14 years, and 4.4% in people aged 15–29 years (Cui et al., 2017). Furthermore, HBV infection was caused by maternal transmission in 30%–50% cases (Onakewhor et al., 2013; Thio et al., 2015). In 2015, a nationwide program in China was implemented, aiming at interrupting mother‐to‐child transmission of HBV, human immunodeficiency virus, and syphilis (Wang et al., 2015). Approaches to prevent mother‐to‐child transmission of HBV include vaccine immunoprophylaxis and administration of hepatitis B immunoglobulin to babies born to mothers infected with the virus (Chamroonkul & Piratvisuth, 2017). For mothers with a high viral load, it is also recommended that antiviral drugs should be administered during the third trimester of pregnancy (Chamroonkul & Piratvisuth, 2017). Previous studies have reported that antiviral therapy with tenofovir can substantially reduce perinatal transmission of HBV in mothers with high viral load (Greenup et al., 2014; Hyun et al., 2017; Jourdain et al., 2016, 2018; Thilakanathan et al., 2018;) and is a cost‐effective treatment (Wang, Wang, et al., 2016). Jourdain et al. reported that MTCT is rare when passive/active immunization is done timely (Jourdain et al., 2018). Similarly, telbivudine has also been reported to greatly inhibit mother‐to‐child transmission of HBV when maternal viral load is high (Han et al., 2011; Lu et al., 2014; Pan et al., 2012; Wu et al., 2015).

Several genotypes of HBV have been described, it is found that various genotypes show different responses to therapy (Lin & Kao, 2011; Tian & Jia, 2016). A small number of studies have suggested that the response to tenofovir may be better for genotype A (Hossain & Ueda, 2019; Marcellin et al., 2014; Zoulim et al., 2015) and genotype D (Marcellin et al., 2014), compared with other genotypes, but worse for genotype G (Hossain & Ueda, 2019). In patients receiving telbivudine monotherapy, subgenotype C1 was associated with a better antiviral response than subgenotypes B2 and C2 (Shen et al., 2018). Interestingly, genotype switching has also been reported in patients with CHB receiving tenofovir, while genotype D is less likely to undergo switching than genotype A or mixed genotypes (Chauhan et al., 2016). Nonetheless, there remains a paucity of data regarding the effects of HBV genotype on the response to therapy with telbivudine or tenofovir, particularly in pregnant women. Because of the special nature of the HBV life cycle, replication, transcription, and expression are dependent on mRNA, as a necessary condition for further replication and expression of corresponding HBV proteins, while mRNA is also a prerequisite for the production of viral particles with infectious activity. Therefore, the aim of this study was to investigate whether HBV genotype was associated with the effects of tenofovir and telbivudine on HBV DNA and RNA levels in HBsAg‐positive pregnant women.

## 2 METHODS

### 2.1 Patients

This was a retrospective study. HBsAg‐positive and hepatitis B e antigen (HBeAg)‐positive pregnant women attending the Affiliated Hospital of Guizhou Medical University (Guizhou, China) between May 2016 and July 2017 were included. The inclusion criteria were pregnant women aged 18–45 years; HBsAg and HBeAg positivity detected at ≤12 weeks of pregnancy; HBV DNA ≥10^6^ IU/mL received antiviral therapy with telbivudine or tenofovir during 24–28 weeks of pregnancy; liver function indexes, HBsAg, HBeAg, HBV DNA, and HBV RNA were measured at 12–24 weeks, 28–32 weeks, and 36–40 weeks of pregnancy. The diagnosis was consistent with the 2015 update of the Guideline of Prevention and Treatment for Chronic Hepatitis B in China (Hou et al., 2017).

The exclusion criteria were interferon therapy received during the 6 months before pregnancy; decompensated liver cirrhosis; and high level of alcohol consumption long‐term (≥20 g alcohol/day for ≥5 years). Patients with history of chronic diseases such as liver disease, diabetes, thyroid disease, hypertension, chronic kidney disease, cancer, or psychiatric illness and patients receiving other drugs during pregnancy, such as immunomodulators, cytotoxic drugs, or corticosteroids were also excluded. Patients with HIV infection were excluded. This study was approved by the ethics committee of our hospital and was performed in accordance with the relevant provisions of the Helsinki Declaration.

### 2.2 Baseline information collection

We reviewed the medical records to collect the following baseline information at 12–24 weeks of pregnancy, including age, time of diagnosis of HBV infection, family history of hepatitis B, liver function indexes (serum levels of alanine transaminase [ALT], aspartate transaminase [AST], total bilirubin [TBIL], total bile acids [TBA], cholinesterase [CHE], alkaline phosphatase [ALP], and gamma‐glutamyl transferase [GGT]), HBsAg, HBeAg, HBV DNA, HBV RNA, and HBV genotype.

### 2.3 Patients’ treatment

With the patient's consent, oral antiviral therapy with telbivudine (Beijing Novartis Pharma Co. Ltd, Beijing, China; approval no.: Chinese medicine H20070028] or tenofovir disoproxil fumarate (GlaxoSmithKline [Tianjin] Co. Ltd, Tianjin, China; approval no.: Chinese medicine H20153090) was initiated at 24–28 weeks of pregnancy. Measurements of liver function indexes, HBsAg, HBeAg, HBV DNA, and HBV RNA were repeated measured 4 weeks after initial antiviral therapy (at 28–32 weeks of pregnancy) and before birth (at 36–40 weeks of pregnancy). All patients were treated with telbivudine or tenofovir from 12 weeks of pregnancy and HBV infection to the date of delivery.

### 2.4 Blood sample collection

Blood samples (5 ml each time) were collected at 12–24, 28–32, and 36–40 weeks of pregnancy and stored in an ethylenediaminetetraacetic acid anticoagulated tube. The sample was centrifuged for 6 min at 1,369 × *g* to separate the serum, and 200 μl aliquots of serum were stored at −70°C until use.

### 2.5 Liver function indexes

An ADVIA 2400–2 automatic biochemistry analyzer (Siemens, Munich, Germany) was used to detect serum levels of ALT, AST, TBIL, TBA, CHE, ALP, and GGT, in accordance with the manufacturer's instructions.

### 2.6 Detection of serum HBsAg and HBeAg

A DR6608‐2 time‐resolved immunofluorescence analyzer (DaAn Gene Co. Ltd, Guangzhou, China) and appropriate reagents (Guangzhou Darui Biotechnology Co. Ltd, Guangzhou, China) were used to detect HBsAg and HBeAg. Serum HBsAg <0.4 IU/mL and serum HBeAg <0.5 PEIU/ml were defined as negative. The dynamic range of each quantitative assay was as follows: HBsAg 5–300 IU/ml and HBeAg 0.01–160 PEIU/ml.

### 2.7 Detection of serum HBV DNA

Serum HBV DNA was detected using a 7,500 Real‐Time PCR System (Applied Biosystems, Foster City, CA, USA) and appropriate reagents (DaAn Gene Co. Ltd) (Espy et al., 2006; Speers, 2006). The sensitivity was 30 IU/ml, the within‐run coefficient of variation (CV) was ≤5%, and the between‐run CV was ≤5%. HBV DNA <100 IU/ml was reported as negative, and the linear detection range was between 100 IU/mL and 5.0 × 10^8^ IU/ml.

### 2.8 Detection of serum HBV RNA

The detection of HBV RNA was performed as previously described (Wang, Shen, et al., 2016). The diagnostic kit for HBV pregenomic RNA (pgRNA; PCR‐Fluorescence Probing) and reagents (batch number: 20170701) were sourced from Hotgen Biotechnology Co. Ltd (Beijing, China). The sensitivity of the assay was 300 copies/mL, the within‐run CV was ≤5%, and the between‐run CV was ≤5%. The results were expressed as logarithms.

### 2.9 HBV genotype detection

HBV genotypes were detected using genotyping kits and accompanying reagents (batch number: 20,170,801; Hotgen Biotechnology Co. Ltd), as described previously (Jin et al., 2008). Genotype A was 709 bp, genotype B was 308 bp, subgenotype C1 was 510 bp, subgenotype C2 was 195 bp, and genotype D was 671 bp.

#### 2.9.1 Statistical analysis

Statistical analyses were performed using SPSS 22.0 (IBM Corp., Armonk, IL, USA). Normally distributed continuous variables were presented as mean ±standard deviation and compared between groups by independent samples *t* test. Non‐normally distributed variables were presented as median (minimum and maximum) and compared between groups by independent samples Mann–Whitney *U* test or Wilcoxon signed‐rank test, as appropriate. Categorical variables were analyzed using Fisher's exact test or the chi‐squared test, as appropriate. Multiple linear regression analyses were performed using a model in which the dependent variable was the HBV DNA change (log_10_ transformed) or the HBV RNA change (log_10_ transformed), and the independent variables were antiviral therapy group (telbivudine vs. tenofovir) and genotype (B vs. C). Odds ratios (ORs) and 95% confidence intervals (95%CIs) were calculated. A two‐sided *p* value < .05 was regarded as statistically significant.

## 3 RESULTS

### 3.1 Baseline characteristics

A total of 74 patients were enrolled in this study. There were 37 patients of telbivudine group and tenofovir group respectively. The baseline characteristics of the study participants were shown in Table 1. The HBV genotype was B in 64.9% of the participants and C in 35.1%. There were no differences between the telbivudine and tenofovir groups in any of the baseline characteristics, including liver function tests, HBV genotype, HBsAg, HBeAg, log_10_(HBV DNA), and log_10_(HBV DNA) (*p* > .05).

**TABLE 1:** Baseline characteristics

Characteristic	Tenofovir (*n* = 37)	Telbivudine (*n* = 37)	*p* value
Age (years), mean ± *SD*	26.22 ± 3.95	27.62 ± 4.97	0.182
Time since diagnosis of HBV infection (years), median (min, max)	5 (0, 25)	3 (0, 29)	0.343
Family history of hepatitis B, *n* (%)			0.244
Yes	22 (59.46)	17 (45.95)	
No	15 (40.54)	20 (54.05)	
Ethnicity, *n* (%)			0.148
Han	27 (72.97)	32 (86.49)	
Non‐Han	10 (27.03)	5 (13.51)	
Nulliparous, *n* (%)	37 (100)	37 (100)	
History of abortion, *n* (%)	0	0	
Genotype, *n* (%)			0.330
B	26 (70.27)	22 (59.46)	
C	11 (29.73)	15 (40.54)	
Laboratory investigations			
ALT (U/L), median (min, max)	24.1 (9.66, 362.49)	19.74 (6.65, 92.04)	0.113
AST (U/L), median (min, max)	24.8 (13.62, 345.53)	24.82 (13.81, 77.77)	0.725
TBIL (µmol/L), median (min, max)	7.35 (3.79, 57.55)	8.2 (4.85, 13.65)	0.940
TBA (µmol/L), median (min, max)	2.75 (0.83, 45.23)	4.13 (1.06, 25.50)	0.077
CHE (U/L), mean ± *SD*	5,858.80 ± 1,408.77	5,756.95 ± 1656.14	0.777
ALP (U/L), median (min, max)	70.15 (31.00, 232.52)	76.31 (46.05, 388.21)	0.534
GGT (U/L), median (min, max)	13.8 (7.12, 79.75)	16.49 (6.71, 41.56)	0.261
HBsAg (IU/L), median (min, max)	290.92 (26.24, 300.00)	285.73 (150.7, 300.00)	0.615
HBeAg (PEIU/L), median (min, max)	160 (7.40, 160.00)	160 (5.82, 160.00)	0.460
Log_10_(HBV DNA), median (min, max)	7.5 (3.3, 8.7)	7.4 (4.7, 8.7)	0.347
Log_10_(HBV RNA), median (min, max)	6.9 (4.1, 8.7)	7.0 (4.1, 8.7)	0.646

Abbreviations: ALP, alkaline phosphatase; ALT, alanine transaminase; AST, aspartate transaminase; CHE, cholesterase; GGT, gamma‐glutamyl transferase; HBeAG, hepatitis B e antigen; HBsAG, hepatitis B surface antigen; HBV, hepatitis B virus; *SD*, standard deviation (*SD*); TBA, total bile acids; TBIL, total bilirubin.

### 3.2 Effects of telbivudine and tenofovir on laboratory parameters

The results of laboratory investigations for the telbivudine and tenofovir groups at 12–24 weeks (baseline), 28–32 weeks, and 36–40 weeks were presented in Table 2. Compared with the respective baseline values, the telbivudine group showed a significant increase in ALP (*p* < .001) and significant reductions in HBsAg (*p* < .05), HBeAg (*p* < .05), log_10_(HBV DNA) (*p* < .001), and log_10_(HBV RNA) (*p* < .001) at 36–40 weeks. The tenofovir group also exhibited a significant increase in ALP (*p* < .001) and significant reductions in HBeAg (*p* < .05), log_10_(HBV DNA) (*p* < .001), and log_10_(HBV RNA) (*p* < .001) at 36–40 weeks, compared with baseline.

**TABLE 2:** Effects of telbivudine and tenofovir on the laboratory parameters of pregnant women with hepatitis B

Drug	Time		ALT	AST	TBIL	ALP	CHE	TBA	GGT	HBsAg	HBeAg	log_10_ (HBV DNA)	log_10_ (HBV RNA)
Telbivudine	T1	median	19.74	24.82	8.20	76.31	6,001.69	4.13	16.49	285.73	160.00	7.43	6.96
		mix	6.65	13.81	4.85	46.05	534.07	1.06	6.71	150.70	5.82	4.68	4.15
		max	92.04	77.77	13.65	388.21	9,785.10	25.50	41.56	300.00	160.00	8.70	8.72
	T2	median	19.62	26.50	8.13	120.30	6,064.80	5.20	17.20	270.30	160.00	4.09	6.30
		mix	8.98	10.67	0.30	49.40	3,741.90	0.72	2.40	204.70	0.88	2.00	3.27
		max	119.97	93.90	16.20	290.93	8,952.60	36.00	40.95	300.00	160.00	7.00	7.89
	T3	median	21.30	23.70	7.71	150.70	6,053.69	5.09	17.40	271.30	160.00	3.38	5.86
		mix	8.14	11.79	4.83	47.30	3,394.96	1.08	6.20	129.29	0.13	2.00	3.91
		max	71.31	160.64	16.21	376.24	9,585.02	30.17	86.19	300.00	160.00	7.30	6.86
*P* (T1 versus. T3)*			0.946	0.827	0.223	**<0.001**	0.060	0.377	0.683	**0.028**	**0.044**	**<0.001**	**<0.001**
Tenofovir	T1	median	24.10	24.80	7.35	70.15	5,821.30	2.75	13.80	290.92	160.00	7.52	6.87
		mix	9.66	13.62	3.79	31.00	3,354.73	0.83	7.12	26.24	7.40	3.32	4.05
		max	362.49	345.53	57.55	232.52	9,658.43	45.23	79.75	300.00	160.00	8.70	8.64
	T2	median	21.10	24.50	6.98	107.70	5,674.04	3.70	14.00	286.34	160.00	4.09	6.45
		mix	8.91	15.20	2.45	4.64	3,612.00	0.63	5.59	19.80	3.20	2.00	2.48
		max	305.90	186.10	19.10	276.60	8,346.10	101.20	219.17	300.00	160.00	6.27	7.87
	T3	median	21.30	24.50	8.60	147.01	5,821.76	3.80	15.51	280.00	160.00	2.98	5.76
		mix	8.76	17.21	3.16	53.98	2,528.00	1.17	5.60	27.40	0.00	2.00	2.48
		max	98.10	101.92	18.40	470.61	9,643.61	31.50	45.95	300.00	160.00	5.01	6.89
*P* (T1 versus. T3)*			0.213	0.402	0.656	**<0.001**	0.572	0.233	0.694	0.285	**0.014**	**<0.001**	**<0.001**
*P* [telbivudine △(T1‐T3) versus. tenofovir △(T1‐T3)]#			0.307	0.570	0.646	0.350	0.970	0.970	0.957	0.330	0.816	**0.048**	0.709

Abbreviations: ALP, alkaline phosphatase; ALT, alanine transaminase; AST, aspartate transaminase; CHE, cholesterase; GGT, gamma‐glutamyl transferase; HBeAG, hepatitis B e antigen; HBsAG, hepatitis B surface antigen; HBV, hepatitis B virus; *SD*, standard deviation (*SD*); T2, 28–32 weeks; T3, 36–40 weeks; TBA, total bile acids; TBIL, total bilirubin. T1, 12–24 weeks.

*Wilcoxon signed‐rank test; # independent samples Mann–Whitney *U* test.

### 3.3 HBV genotype was independently associated with HBV DNA change after therapy with telbivudine or tenofovir

HBV genotype (B vs. C) was a factor independently associated with HBV DNA change after therapy (*p* = .005) but not with HBV RNA change after therapy (*p* = .096). The type of drug used (telbivudine versus. tenofovir) was not associated with HBV DNA change or HBV RNA change after therapy (Tables 3 and 4).

**TABLE 3:** Multivariate linear regression analysis of the factors associated with hepatitis B virus DNA change (log_10_ transformed)

Factor	β coefficient	SE	*p* value
Group (tenofovir versus. telbivudine)	0.484	0.287	0.096
Genotype (B versus. C)	−0.868	0.300	**0.005**

Note

SE: standard error of the β coefficient.

**TABLE 4:** Multivariate linear regression analysis of the factors associated with hepatitis B virus RNA change (log_10_ transformed)

Factor	β coefficient	SE	*p* value
Group (tenofovir versus. telbivudine)	−0.074	0.206	0.721
Genotype (B versus. C)	−0.364	0.216	0.096

Note

SE: standard error of the β coefficient.

## 4 DISCUSSION

Molecular epidemiologic studies reveal remarkable differences in the geographical distribution of HBV genotypes. The frequency of mutants among HBV genotypes also varies. The role of HBV genotypes/mutants in the pathogenesis of HBV infection and natural history of HBV infection has been extensively investigated. The distribution of HBV genotypes in acute hepatitis B patients reflects the predominant genotypes in a given geographic area. Genotypes B and C are the most common type in Asia‐Pacific region. In the present study, HBV genotyping in 74 pregnant women with HBeAg‐positive hepatitis B in Guizhou of China revealed that there were 48 cases (64%) of genotype B and 27 cases (35%) of genotype C, and no other genotypes were found. It indicated that the HBV genotypes in pregnant women with hepatitis B in Guizhou were mainly B and C, which was consistent with the results of previous studies on pregnant women in China (Ding et al., 2013; Guo et al., 2002; Kang et al., 2017; Yin et al., 2016).

HBV is a kind of hepatovirus that involves the liver, hence liver function measurement is often used to reflect whether the liver has been affected by HBV infection. There have been conflicting data regarding the relationship between HBV genotype and liver function. Some studies have showed that genotype C‐infected patients may have a higher HBeAg level and be more likely to develop liver damage and cirrhosis than genotype B‐infected patients (Guo et al., 2016; Lin & Kao, 2011; Xibing et al., 2013), whereas another research has reported that lifetime spontaneous loss of HBsAg is more common for genotype C than genotype B (Tseng et al., 2015).

The present study did not directly compare the results of liver function between pregnant women with genotype B infection and those with genotype C infection. However, our analysis showed that there were no significant differences in ALT, AST, TBIL, ALP, CHE, TBA, or GGT between the telbivudine and tenofovir groups at any time points. One possible explanation should be that, due to the action of progestin, the pregnant women in our study were in a state of immunosuppression with no significant abnormalities in liver function, even in cases with a high viral load. In addition, we found only minimal changes in HBsAg and HBeAg during therapy with telbivudine or tenofovir. It indicated that the short‐term effects of nucleos(t)ide analogues on HBsAg and HBeAg during pregnancy were small, which were consistent with the characteristics of HBV infection/replication and the results of previous studies (Li et al., 2014; Na et al., 2021; Tseng & Kao, 2013; Zoutendijk et al., 2011).

The use of telbivudine or tenofovir caused significant reductions in HBV DNA and HBV RNA, but there were no significant differences in antiviral effects between the two drugs. Moreover, in our study, maternal liver function was stable and viral control was good during pregnancy, there were no preterm delivery or abortion occurred in our study, and the Apgar scores of the newborns were also normal after birth. Therefore, we concluded that the use of telbivudine or tenofovir after 24 weeks of pregnancy was safe and effective, which was similar to the results of previous studies (Greenup et al., 2014; Han et al., 2011; Hyun et al., 2017; Jourdain et al., 2016; 2018; Lu et al., 2014; Pan et al., 2012; Thilakanathan et al., 2018; Wu et al., 2015).

HBV genotype was found to be an independent factor associated with the response of HBV DNA level to therapy in our study, which indicated that there might be also differences in prognosis, including mother‐to‐child transmission, between genotypes B and C. There was some, albeit limited, evidence showing that the response to treatment might vary between different genotypes (Lin & Kao, 2011; Tarao et al., 2021; Tian & Jia, 2016). It has been suggested that genotypes A and possibly D showed a better response to tenofovir (Hossain & Ueda, 2019; Zoulim et al., 2015), whereas genotype G showed a poorer response (Marcellin et al., 2014). Furthermore, subgenotype C1 appeared to exhibit a better response to telbivudine than subgenotypes B2 and C2 (Shen et al., 2018). Our current study found that the response to nucleos(t)ide analogues might differ between genotypes B and C, which might possibly lead to differences in the risk of mother‐to‐child transmission. This novel founding on the maternal HBV genotype might facilitate stratification of the risk of mother‐to‐child transmission of HBV in China, where the main genotypes were B and C. In our study, therapy with telbivudine or tenofovir led to a notable decrease in HBV RNA, which would be consistent with a virologic response. However, HBV genotype showed no association with the response of HBV RNA to therapy, suggesting that nucleos(t)ide analogues have similar effects on HBV RNA for genotypes B and C. HBsAg comes from either cccDNA or integrated gene fragments. HBsAg cannot completely represent the transcription activity of HBV cccDNA. HBV RNA, also known as pgRNA, only comes from cccDNA and can accurately reflect the cccDNA level. With the comprehensive knowledge on HBV RNA, the use of simultaneous continuous clearance of serum HBV DNA and HBV RNA is suggested as the safe stopping rule in patients with CHB on NAs treatment.

There were also some limitations in this study. First, this was a retrospective analysis; therefore, the selection and reporting bias cannot be excluded. Second, this was a single‐center study. Third, our sample size was quite small. Fourth, the effect of genotype on mother‐to‐child transmission of HBV was not assessed. Fifth, the influence of genotype on longer‐term outcomes was not investigated. Therefore, the evidence of this study is still relatively insufficient, further prospective, multicenter studies with larger sample sizes are needed to extend our findings. The increase in ALP during therapy is worth our attention; however, in this study, there is no control group. The mechanism behind the increase in ALP is still unclear. Further study to evaluate the changes in ALP is needed.

In conclusion, HBV genotype was independently associated with the response of serum HBV DNA level change to therapy with telbivudine or tenofovir in pregnant women with hepatitis B in Guizhou. It seems that genotype B is easier to be treated than genotype C. Tenofovir is slightly more effective than telbivudine. These findings may help risk assessment regarding vertical transmission of HBV in HBeAg‐positive mothers treated with nucleos(t)ide analogues.

## AUTHOR CONTRIBUTIONS

**Mingliang Cheng, Baofang Zhang:** guarantor of integrity of the entire study. **Lei Yu, Baofang Zhang:** study concepts. **Mingliang Cheng, Quan Zhang:** study design. **Lei Yu, Xueke Zhao:** definition of intellectual content. **Baofang Zhang, Jun Wu:** literature research. **Shuang Lu, Jing Yang, KaiSheng Deng:** clinical studies. **Yongmei Liu, Jun Wang, Peiling Zhao:** experimental studies. **Baofang Zhang, Peiling Zhao:** data acquisition. **Baofang Zhang, KaiSheng Deng:** data analysis. **Lei Yu, Yongmei Liu:** statistical analysis. **Mingliang Cheng, Baofang Zhang:** manuscript preparation. **Lei Yu, Xueke Zhao:** manuscript editing. **Mingliang Cheng, Baofang Zhang, Quan Zhang:** manuscript review.

## CONFLICT OF INTEREST

The authors declare that they have no conflicts of interest.

## ETHICAL APPROVAL

This study was approved by the ethics committee of our hospital and was performed in accordance with the relevant provisions of the Helsinki Declaration. Written consent was obtained.

## CONSENT FOR PUBLICATION

All patients signed informal consent.

## CONSENT TO PARTICIPATE

All authors have read and approved the content, and agree to submit for consideration for publication in the journal.

## Data Availability

This data and materials are available.
